# The mediating role of coping styles in the relationship between personality traits and occupational well-being among nursing staff

**DOI:** 10.3389/fpubh.2025.1642906

**Published:** 2025-09-03

**Authors:** Fei Wang, Sheng Li, Wenjie Liu, Yifei Li, Qing Jia, Jinyu Wang

**Affiliations:** ^1^School of Public Health, Gansu University of Chinese Medicine, Lanzhou, China; ^2^The No.2 People’s Hospital of Lanzhou, Lanzhou, China; ^3^First School of Clinical Medicine, Lanzhou University, Lanzhou, China; ^4^LanZhou Pulmonary Hospital, Lanzhou, China; ^5^School of Public Health, Lanzhou University, Lanzhou, China

**Keywords:** nursing staff, personality traits, coping styles, occupational well-being, mediating role

## Abstract

**Objective:**

This study aimed to quantify the mediating effects of positive and negative coping styles on the relationship between the Big Five personality traits and occupational well-being among nurses. The findings aim to provide a scientific basis for optimizing psychological health interventions for nurses.

**Methods:**

A cross-sectional study was conducted, involving 9,578 nursing staff from over 50 hospitals of varying tiers in Lanzhou, China. Standardized scales—the Chinese Big Five Personality Inventory-Brief (CBF-PI-B), Simplified Coping Style Questionnaire (SCSQ), and Occupational Well-being Scale for Healthcare Workers—were administered. Mediation effects were tested using the Bootstrap method, with adjustments for covariates including gender, age, and department.

**Results:**

Personality traits showed significant correlations with occupational well-being: Extraversion, conscientiousness, agreeableness, and openness were positively correlated with well-being (*r* = 0.337 to 0.400), while neuroticism was negatively correlated (*r* = −0.338). Coping styles played a pivotal mediating role: Neuroticism indirectly reduced well-being through negative coping strategies (e.g., problem avoidance), while conscientiousness, agreeableness, openness, and extraversion correlated with positive coping strategies (e.g., active problem-solving) and higher well-being. The mediating effect of positive coping was substantially larger, accounting for 23.69 to 31.93% of the total effects, whereas negative coping accounted for less than 5.69%.

**Conclusion:**

Personality traits indirectly affect occupational well-being via positive or negative coping strategies, with proactive coping serving as the critical pathway for well-being enhancement. This study reveals an asymmetry in the mediating mechanism, where the efficacy of positive coping far outweighs that of negative coping. These insights offer novel perspectives for developing targeted interventions, such as personality assessment-guided coping skills training. These findings support a ‘Coping Efficacy Asymmetry Model’ providing a new framework for interventions that prioritize building positive coping skills to enhance nurse well-being and support healthcare system resilience.

## 1 Introduction

Occupational well-being is defined as employees’ subjective evaluation of their work satisfaction, reflecting their sense of contentment and happiness in their jobs (1). It emphasizes the balance between subjective perceptions and objective adaptability to work demands, serving as a critical indicator of nursing staff’s psychological health and professional adaptability (2). Notably, occupational well-being is not only linked to nurses’ quality of life but is also closely associated with the quality of patient care and the stability of the healthcare system (2). However, nursing staff, as the core of healthcare delivery, face persistent challenges: heavy workloads, complex medical environments, high occupational risks, and low social recognition. This high-stress environment contributes to a high prevalence of emotional exhaustion and burnout, which compromises nurses’ occupational well-being and, consequently, patient care quality (3). Addressing these issues requires clarifying the underlying mechanisms to develop targeted interventions.

Coping styles refer to the cognitive and behavioral strategies individuals adopt to manage stressors, which can be categorized into positive coping (e.g., problem-solving, seeking social support) and negative coping (e.g., self-blame, avoidance) (4). Previous studies have shown that positive coping enhances psychological flexibility and mitigates mental fatigue, while negative coping exacerbates distress (5). For nursing populations, coping styles are particularly critical: Yeh et al. found that coping strategies significantly correlate with psychological well-being among nursing students across cultures, highlighting their cross-contextual relevance (6).

Personality traits, as stable psychological characteristics, are key predictors of occupational behaviors and mental health (7). The Big Five personality framework (conscientiousness, neuroticism, openness, agreeableness, and extraversion) has been widely used to explore individual differences in nursing staff (8). Prior research has confirmed direct associations between personality traits and occupational well-being: for example, conscientiousness and extraversion are positively correlated with well-being, while neuroticism is negatively correlated (9). Additionally, coping styles have been identified as a mediator in the relationship between personality and well-being—Lu et al. demonstrated that coping styles mediate the effect of perfectionism on subjective well-being, supporting the potential of such pathways (10).

However, two critical gaps persist in the literature. First, few studies have simultaneously modeled the mediating pathways of both positive and negative coping styles within a single framework. Second, the comparative efficacy of these pathways—whether proactive strategies are more influential than the avoidance of negative ones—has not been quantified in a large nursing cohort. This study addresses these gaps directly, especially the chained mediation of “personality traits → coping styles → occupational well-being” among nursing staff (11). The concepts of “occupational well-being” and “coping styles” lack operationalization within a clear theoretical framework for nursing populations, and their interplay with personality has not been fully explored (12). Meanwhile, little is known about the buffering role of personality and coping styles in the impact of nursing-specific workplace stressors (13). Against this background, this study aims to systematically explore the intrinsic relationships among personality traits, coping styles, and occupational well-being in nursing staff, with a focus on analyzing the mediating role of coping styles. Specifically, we hypothesize that:

*H*1: Personality traits (extraversion, conscientiousness, agreeableness, openness) are positively correlated with occupational well-being, while neuroticism is negatively correlated.

*H*2: Positive coping styles mediate the positive effects of extraversion, conscientiousness, agreeableness, and openness on occupational well-being.

*H*3: Negative coping styles mediate the negative effect of neuroticism on occupational well-being.

By addressing these hypotheses, this study aims to fill theoretical gaps, provide a theoretical basis for optimizing mental health interventions for nursing professionals, and offer empirical evidence for enhancing their occupational well-being and healthcare system stability.

## 2 Materials and methods

### 2.1 Study participants

This study employed a cross-sectional design conducted in Lanzhou City from February to March 2025. To enhance sample representativeness, we implemented a stratified convenience sampling strategy by categorizing all hospitals into three tiers based on accreditation levels (tertiary, secondary, and primary). Over 50 hospitals that met the criteria, including high compliance, operational feasibility, and multi-tier coverage, were selected as participating institutions. It should be noted that the cross-sectional nature of the data inherently precludes causal-temporal inferences, constituting a fundamental methodological constraint of this investigation. The inclusion criteria were: (1) one year or more of work experience; (2) Licensed in-service nurses; (3) No history of serious physical illnesses; and (4) Voluntary participation are requirements for inclusion. Exclusion criteria included nurses in training programs, interns, rehired retired nurses, and nurses on maternity leave or breastfeeding. 9,578 nursing staff members were recruited using a multi-stage sampling technique. An effective response rate of 91.79% was obtained by keeping 8,793 valid surveys after excluding those with glaring logical mistakes or missing answers. This study has been approved by the Ethics Committee of Lanzhou First People’s Hospital (No. 2025A-8).

### 2.2 Survey instruments

#### 2.2.1 General information questionnaire

After a thorough analysis of pertinent literature, the General Information Questionnaire was created. Employing institution, gender, age, department, position, professional title, greatest educational attainment, marital status, years of work experience, monthly pay, and other pertinent factors were among the 16 questions that covered demographic and occupational characteristics.

#### 2.2.2 Chinese big five personality inventory brief version (CBF-PI-B)

Conscientiousness, neuroticism, openness, agreeableness, and extraversion are the five main dimensions covered by the 40 items on this scale, which was created by Mengcheng Wang and Xiaoyang Dai and is based on the Chinese Big Five Personality Inventory framework. Each dimension has eight items. With internal consistency coefficients and test–retest reliability that satisfy accepted psychometric standards, the scale has strong psychometric qualities and uses a 6-point Likert scoring system (1 = “Strongly Disagree,” 6 = “Strongly Agree”) (8). Dimension Definitions: In goal-directed activities, conscientiousness is a reflection of self-control, organization, and a sense of accountability. While lower scores imply impulsivity and inconsistency, higher scores show greater perseverance, dependability, and accomplishment orientation. The alpha coefficient for this dimension is 0.802. Emotional stability is measured by neuroticism (with an alpha coefficient of 0.83). High scores on this dimension indicate a greater propensity for emotional instability, including anxiety and negative affect, whereas low scores reflect higher emotional stability. Openness assesses a person’s propensity for cognitive exploration. High scorers embrace new experiences and autonomous judgment, exhibiting open-mindedness, creative thinking, and esthetic sensibility. Individuals with low scores exhibit strict behavioral patterns and cognitive conservatism. The alpha coefficient for this dimension was 0.825. Agreeableness evaluates how people connect with a Cronbach’s alpha coefficient of 0.748. Low scorers exhibit competitive and cynical inclinations, whereas high scorers are cooperative, sympathetic, and selfless. Extraversion studies how people interact with others, with this dimension’s Cronbach’s alpha coefficient being 0.694. In contrast to the restrained and introverted characteristics of low scorers, high scorers are gregarious, energetic, and positively emotional. The CBF-PI-B is a validated assessment instrument with a total alpha coefficient of 0.849 for examining the connections between personality traits and occupational behaviors because it successfully distinguishes individual differences across these five fundamental personality traits through standardized scoring processes.

#### 2.2.3 Simplified coping style questionnaire

Yaning Xie and colleagues’ internationally acclaimed coping style inventories served as the cultural basis for this scale, which has 20 self-rated items with a 4-point Likert scale (0 = “Never use” to 3 = “Frequently use”) (4). It uses a two-factor framework: Higher scores on the Positive Coping (items 1–12) scale indicate proactive coping tendencies. This scale measures positive tactics, such as problem-solving and seeking social assistance. Items 13–20 measure negative coping, which evaluates maladaptive behaviors including self-blame and avoidance. Higher scores indicate more robust passive coping strategies. Item scores within each dimension are added up to determine scores. Subscale scores that contrast provide a methodical examination of people’s preferred coping mechanisms. This scale has demonstrated good reliability and validity in domestic studies. Specifically, the Cronbach’s alpha coefficients were 0.914 for the positive coping dimension and 0.794 for the negative coping dimension, with an overall scale alpha coefficient of 0.881. It effectively distinguishes the characteristics of coping strategies among different populations.

#### 2.2.4 Occupational well-being scale for healthcare workers

Dongmei Hu’s (14) Occupational Well-Being Scale for Healthcare Workers served as the model for the scale utilized in this investigation. It consists of 24 measures spread over five dimensions: Work Environment (4 items), Financial Compensation (3 items), Social Support (5 items), Sense of Competence/Value Realization (6 items), and Physical and Mental Health (6 items), with the Cronbach’s alpha coefficients for each dimension being 0.877, 0.927, 0.905, 0.894, and 0.896, respectively. A 5-point Likert scale, with 1 denoting “Completely Disagree” and 5 denoting “Completely Agree,” was used to rate the items; higher scores indicated stronger agreement. Greater occupational well-being is indicated by higher overall scores. To conform to the general scoring direction, the responses to the six reverse-scored items on the Physical and Mental Health subscale are inverted (for example, 5 → 1, 4 → 2) before summation.

### 2.3 Data collection and quality control methods

Data was gathered electronically using Wenjuanxing, a popular online survey platform in China. The research team worked with ward head nurses to organize systematic training sessions after receiving official approval from the nursing departments of participating institutions. The study’s goals, evaluation criteria, academic relevance, and operating procedures were all made clearer throughout these meetings. Nursing departments systematically recruited nurses through departmental rosters to minimize volunteer bias. A special focus was on maintaining participant confidentiality and anonymity while closely following the established inclusion/exclusion criteria. After gaining informed consent, head nurses sent nursing staff members questionnaires to complete anonymously. Participants were only allowed to submit once per device and IP address in order to avoid duplicate responses. To ensure data completeness, all survey items were set as mandatory. Every questionnaire was rigorously reviewed once it was collected. Responses showing patterns of inattentive answering (e.g., straight-lining) or completion times under 2 mins were excluded from the final analysis to ensure data quality.

### 2.4 Statistical methods

SPSS 27.0 was used for data analysis. The Shapiro–Wilk test was used to determine whether continuous variables were normal. Continuous data are shown as the median and interquartile range [M (P25, P75)], whereas categorical data are described as frequency and percentage (%), because scores from the Occupational Well-Being Scale for Healthcare Workers, the Chinese Big Five Personality Inventory Brief Version (CBF-PI-B), and the Simplified Coping Style Questionnaire (SCSQ) deviated from normality assumptions. Non-parametric tests were used to evaluate group differences: the Kruskal-Wallis H test for multi-group comparisons and the Wilcoxon rank-sum test for two-group comparisons. To investigate relationships between personality characteristics, coping style, and well-being scores, Spearman’s rank correlation analysis was utilized. With bias-corrected 95% CIs, bootstrap resampling (5,000 iterations) was used to investigate mediation effects using the PROCESS macro v4.1 (15, 16). Two-tailed statistical significance was established at *α* = 0.05.

## 3 Results

### 3.1 Distributions of personality traits, coping styles, and occupational well-being across sociodemographic characteristics

**Figure 1:**
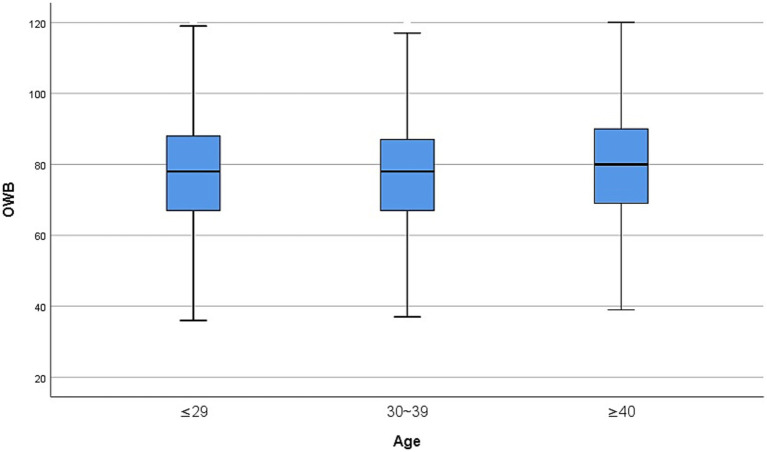
Box plot comparison of occupational well-being by age groups.

Results: Occupational well-being and personality traits exhibited significant disparities across demographic groups (*p* < 0.05), with core trends detailed in Figure 1 and Table 1. Nurses in Class 3B hospitals demonstrated the highest occupational well-being, significantly outperforming those in secondary hospitals (*p* < 0.001), while tertiary hospital nurses displayed elevated neuroticism levels, reflecting the dual impact of high-stress clinical environments on mental health. Nurses aged over 40 exhibited optimal psychological resilience, characterized by the lowest neuroticism, highest conscientiousness, and greatest occupational well-being. Nurses aged over 40 reported the lowest neuroticism, highest conscientiousness, and highest occupational well-being (*p* < 0.001). Emergency department nurses faced the most pronounced challenges, showing the highest neuroticism and lowest well-being (significantly lower than administrative departments, *p* < 0.001), underscoring the direct mental health toll of frontline clinical pressures. Nurses with monthly incomes exceeding 6,000 RMB reported significantly higher occupational well-being (*p* < 0.001), corroborating the salary satisfaction theory that economic security underpins professional fulfillment.

**Table 1:** Personality trait scores, coping styles, and occupational well-being across sociodemographic groups of nursing staff [Median (P25–P75)].

Characteristic		Frequency (n,%)	N	C	PC	NC	OWB
Hospital Tier	Tertiary Grade A	4,712(53.6)	27(22, 31)	33(30, 37)	36(31, 37)	20(16, 24)	79(68, 88)
	Tertiary Grade B	1,144(13)	27(23, 31)	33(30, 38)	36(31, 38)	20(16, 24)	81(68, 90)
	Secondary Grade A	2,196(25)	27(22, 31)	33(30, 37)	36(30, 37)	20(16, 24)	77(66, 88)
	Secondary Grade B	397(4.5)	26(21.5, 31)	32(29, 37)	35(29, 36)	20(16, 24)	74(64, 86)
	Primary	86(1)	25.5(21.75, 29)	33(30, 37)	35(31, 36)	18(15, 23)	77.5(67, 87)
	Unclassified	258(2.9)	27(23, 31)	32(29, 37)	35(29.75, 36)	19(16, 24)	73(62, 84)
	*p*		0.027	0.015	0.001	<0.001	<0.001
Age	≤29	3,056(34.8)	27(22, 31)	32(30, 37)	36(30, 36)	20(16, 24)	78(67, 88)
	30–39	4,234(48.2)	28(23, 31)	33(30, 37)	36(31, 37)	20(16, 24)	78(67, 87)
	≥40	1,503(17.1)	26(21, 30)	35(31, 39)	36(32, 39)	20(16, 23)	80(69, 90)
	*p*		<0.001	<0.001	<0.001	<0.001	0.001
Department	Emergency Department	565(6.4)	27(22.5, 31)	33(30, 37)	35(30, 37)	19(16, 24)	76(67, 86)
	Clinical Departments	7,309(83.1)	27(22, 31)	33(30, 37)	36(31, 37)	20(16, 24)	78(67, 88)
	Paraclinical Departments	768(8.7)	27(23, 31)	33(30, 37)	36(31, 37)	20(17, 24)	81(71, 90)
	Administrative Departments	151(1.7)	25(20, 30)	35(31, 39)	36(32, 38)	19(16, 22)	82(70, 92)
	*p*		0.041	0.02	0.088	0.016	<0.001
Monthly income	<3,000	2,963(33.7)	28(23, 31)	32(29, 37)	35(29, 36)	20(16, 24)	74(63, 85)
	3,000–6,000	4,824(54.9)	27(22, 31)	33(30, 37)	36(31, 37)	20(16, 24)	79(68, 89)
	>6,000	1,006(11.4)	26(20, 30)	35(31, 39)	36(33, 39)	20(16, 24)	83(72, 93)
	*p*		<0.001	<0.001	<0.001	0.017	<0.001
Years of experience in healthcare industry (Years)	≤5	2,540(28.9)	27(22, 31)	32(30, 37)	36(30, 36)	20(16, 24)	79(67, 89)
	6–10	2,704(30.8)	27(23, 31)	32(30, 37)	36(30, 36)	20(16, 24)	76(66, 86)
	11–15	1934(22)	27(23, 31)	33(30, 37)	36(31, 37)	21(17, 24)	78(67, 88)
	16–20	688(7.8)	26(22, 31)	34(31, 38)	36(32, 38)	20(16, 24)	81(69, 90.75)
	21–25	332(3.8)	26(22, 30)	35(31, 39)	36(33, 40)	20(17, 23)	79(68.25, 88.75)
	>25	595(6.8)	25(20, 30)	36(32, 40)	36(33, 39)	20(16, 23)	79(69, 89)
	*p*		<0.001	<0.001	<0.001	<0.001	<0.001

N represents Neuroticism, C represents Conscientiousness, PC represents Positive Coping, NC represents Negative Coping, OWB represents Occupational Well-Being. Nurses in emergency departments exhibited significantly lower well-being scores (Mdn = 76) compared to their counterparts in administrative departments (Mdn = 82, *p* < 0.001), highlighting fundamental disparities in clinical stress exposure.

### 3.2 Correlation analysis of personality traits, coping styles, and occupational well-being

Spearman correlation analysis revealed systematic associations among personality traits, coping styles, and occupational well-being (see Table 2), providing preliminary support for the mediation hypothesis. Occupational well-being showed significant positive correlations with conscientiousness (*r* = 0.400), agreeableness (*r* = 0.339), and openness (*r* = 0.356), while exhibiting a negative correlation with neuroticism (*r* = −0.338; all *p* < 0.01). This indicates that dutifulness, collaborative empathy, and exploratory tendencies enhance professional fulfillment, whereas emotional instability in highly neurotic individuals may amplify stress perception to diminish well-being. These results indicate that conscientiousness, agreeableness, and openness are associated with enhanced professional fulfillment, while emotional instability is associated with diminished well-being. Personality traits significantly predicted coping strategy preferences: Conscientiousness and openness strongly drove active coping, reflecting problem-solving orientation. Neuroticism triggered passive coping while suppressing proactive approaches, highlighting avoidance tendencies. Mechanistically, conscientious and open personalities enhanced well-being through active coping, whereas neuroticism undermined well-being via passive coping through indirect pathways.

**Table 2:** Correlation analysis of personality traits, coping styles, and occupational well-being scores.

Variables	N	C	A	O	E	PC	NC	OWB
N	1							
C	−0.115**	1						
A	−0.245**	0.652**	1					
O	0.083**	0.576**	0.340**	1				
E	−0.104**	0.354**	0.217**	0.633**	1			
PC	−0.150**	0.519**	0.454**	0.492**	0.395**	1		
NC	0.356**	−0.021*	−0.089**	0.220**	0.155**	0.295**	1	
OWB	−0.338**	0.400**	0.339**	0.356**	0.337**	0.342**	−0.054**	1

** represents *p* < 0.01, * represents *p* < 0.05. N represents Neuroticism, C represents Conscientiousness, A represents Agreeableness, O represents Openness, E represents Extroversion, PC represents Positive Coping, NC represents Negative Coping, and OWB represents Occupational Well-Being.

### 3.3 Mediating effects of coping styles in the association between personality traits and occupational well-being

**Figure 2:**
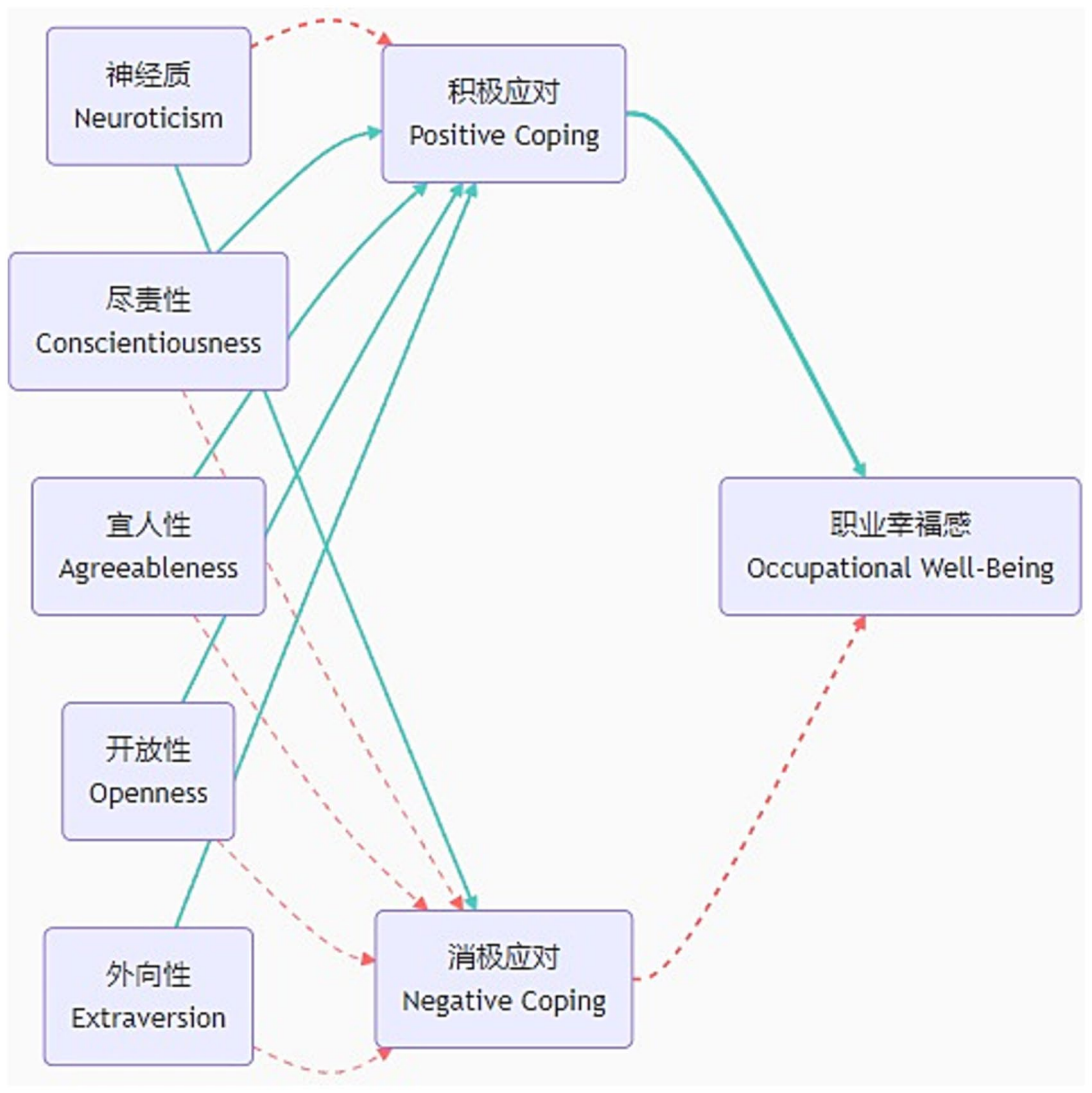
Path diagram of mediation effect analysis.

**Figure 3:**
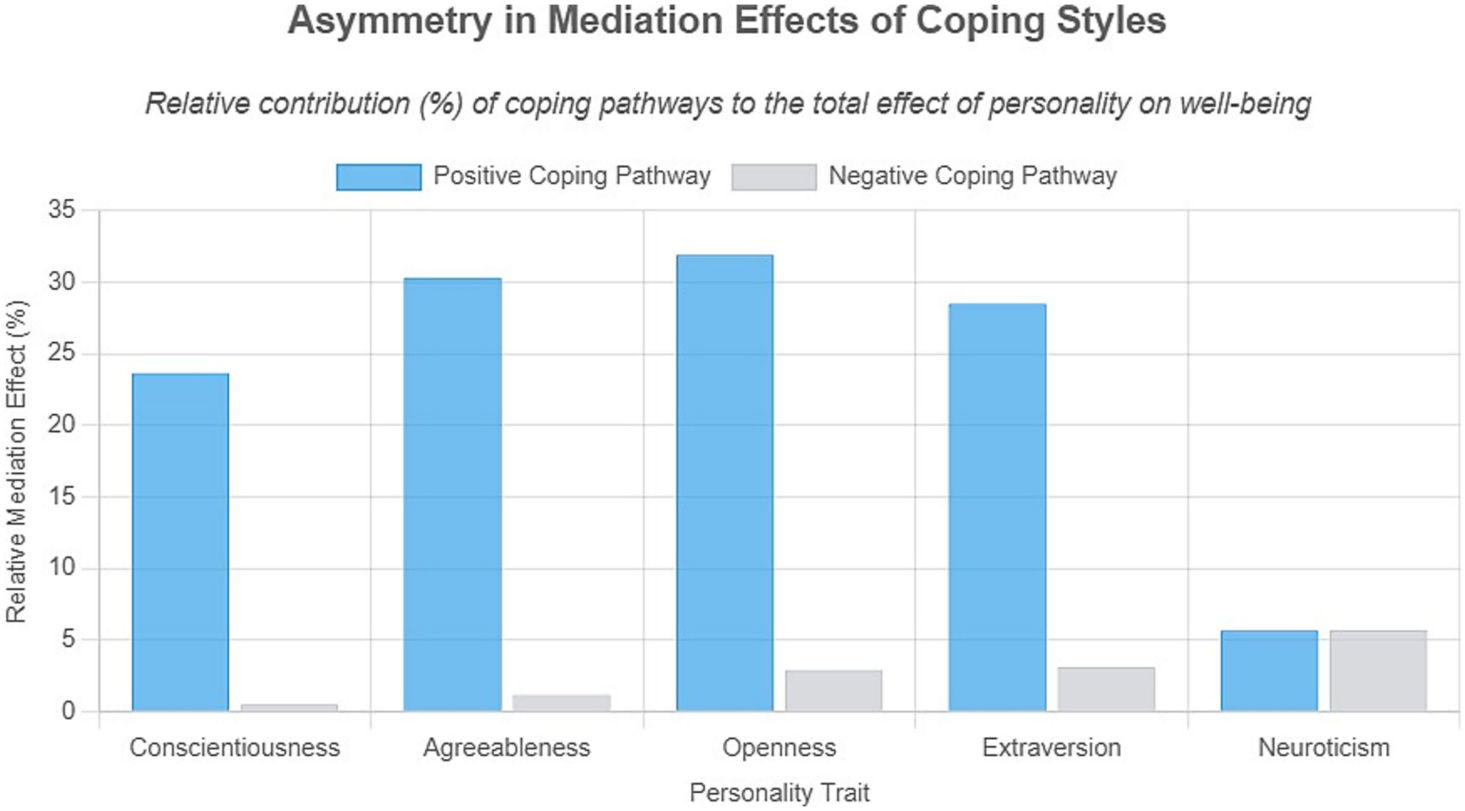
“Asymmetry in Mediation Effects of Coping Styles” shows the relative mediation effect percentages for personality traits. Positive coping pathways (blue) have higher values than negative ones (gray) across Conscientiousness, Agreeableness, Openness, and Extraversion. Neuroticism displays nearly equal values for both pathways.

Bootstrap testing confirmed that coping styles constitute the central mechanism through which personality traits influence occupational well-being. Key findings revealed: Dominant role of active coping: Significant mediating effects were observed between conscientiousness, agreeableness, openness, extraversion, and occupational well-being. These indirect effects accounted for 23.69–31.93% of total effects, with the openness pathway being strongest: 31.93% of openness’s impact was transmitted through “openness → active coping → well-being,” meaning 0.327 units of well-being gain per 1-unit increase in openness derived from optimized coping strategies. Marginal role of passive coping: Weak mediation emerged for neuroticism and select traits (e.g., conscientiousness, agreeableness), contributing <5.69% to total effects. Though the pathway coefficient to well-being was significantly negative (*β* = −0.054*, *p* < 0.05), its explanatory power remained limited, positioning passive coping as a secondary regulatory factor with substantially lower efficacy than active coping (see Table 3; Figures 2, 3).

**Table 3:** Mediation effect analysis of coping styles in the relationship between personality traits and occupational well-being.

Variables	Mediating variable	Indirect effect	Boot SE	Boot 95%CI	Relative mediation effect (ai×bi/c)/%
N	PC	−0.0561	0.0039	−0.0639–−0.0485	5.67
	NC	0.0563	0.0094	0.0377–0.075	5.69
C	**PC**	**0.2952**	**0.0206**	**0.2554**–**0.3365**	**23.69**
	NC	0.0065	0.0026	0.0018–0.012	0.52
A	PC	0.3498	0.0193	0.3104–0.387	30.36
	NC	0.0136	0.0035	0.0073–0.0212	1.18
O	**PC**	**0.3265**	**0.0178**	**0.2911**–**0.3616**	**31.93**
	NC	−0.03	0.0027	−0.0356–−0.0249	2.93
E	PC	0.3141	0.0163	0.283–0.3472	28.52
	NC	−0.0343	0.0051	−0.0447–−0.0249	3.11

N represents Neuroticism, C represents Conscientiousness, A represents agreeableness, O represents openness, E represents extroversion, PC represents Positive Coping, NC represents Negative Coping, and OWB represents Occupational Well-Being. The relative mediation effect, calculated as (Indirect Effect / Total Effect) × 100%, quantifies the proportion of variance explained through mediation pathways. For instance, Openness’s 31.93% value indicates that nearly one-third of its total influence on well-being operates via active coping strategies. Crucially, active coping demonstrated over fivefold greater mediation contributions compared to negative coping across all four major personality traits. Bold values highlight contribution values of core pathways.

## 4 Discussion

This study demonstrates that the influence of personality on nurses’ occupational well-being is primarily mediated by coping styles, with a pronounced asymmetry in efficacy: positive coping strategies are substantially more impactful in promoting well-being than negative coping strategies are in diminishing it. This finding introduces the ‘Coping Efficacy Asymmetry Model’ as a new lens for occupational health. Personality traits do not directly determine occupational well-being but exert indirect effects through dynamic coping strategies in stress contexts. The pivotal finding lies in the significant efficacy asymmetry of mediation pathways: Active coping dominates the relationship between positive traits (e.g., conscientiousness, agreeableness) and well-being, accounting for 23.69–31.93% of total effects. Passive coping exhibits only marginal regulatory effects in neuroticism-related pathways (<5.69%). This asymmetry offers a new lens for understanding nurses’ psychological adaptation mechanisms, emphasizing that cultivating proactive coping strategies—rather than merely mitigating negative responses—is critical for enhancing professional fulfillment.

Interpretation from the Conservation of Resources (COR) Theory (17): The findings gain profound interpretation through the COR lens: individuals persistently seek to acquire and protect valued resources. Positive personality traits such as conscientiousness and openness represent nurses’ stable internal resources. Individuals with these traits predominantly adopt active coping (e.g., proactive problem-solving)—a resource gain strategy. This approach not only mitigates work stress but also facilitates access to new resources (e.g., social recognition, skill enhancement, achievement), thereby forming a resource gain spiral that elevates occupational well-being. As demonstrated by Wu et al.’s research (18), the proactive utilization of character strengths inherently constitutes a process of building psychological resources and enhancing well-being. Conversely, high neuroticism reflects a “susceptibility to resource depletion.” When confronting equivalent work stressors, highly neurotic individuals exhibit heightened threat perception and gravitate toward passive coping strategies (e.g., avoidance, denial). Within the COR framework, this represents a high-cost, low-efficiency resource preservation behavior. While such strategies may provide transient emotional relief, chronic avoidance risks perpetuating—and even exacerbating—stressors. Over time, this culminates in psychological energy depletion and entrenches individuals in a “resource exhaustion trap,” aligning with empirical evidence linking neuroticism to occupational burnout (19).

Our findings engage in critical dialog with the Transactional Theory of Stress: While traditional perspectives emphasize the context-dependent nature of stress appraisal and coping (20), this study reveals that such appraisal and selection are neither entirely stochastic nor context-neutral—they are predominantly shaped by the “presets” of stable personality traits. Crucially, we transcend the dichotomous framework of active/passive coping, demonstrating substantive differences in their efficacy: Active coping exhibits significantly higher pathway coefficients, proving that proactively adopting constructive strategies holds greater transformative potential for well-being enhancement than merely avoiding negative reactions. This asymmetry fundamentally reorients nursing interventions: Efforts should prioritize systematically strengthening nurses’ active coping capacity (e.g., problem-solving training, resilience-building programs) rather than focusing narrowly on suppressing maladaptive responses (21).

This study delivers significant theoretical and practical contributions. Theoretically, it proposes the “Coping Efficacy Asymmetry Model,” which underscores the critical need to differentiate between coping strategies in nurse well-being research. By redefining active coping as the central driver of career adaptability, the study demonstrates that nurses with positive personality traits not only address immediate challenges through effective coping but also cultivate adaptive capacities in practice, thereby enhancing long-term occupational resilience and job satisfaction (22, 23). Practically, the findings provide empirical support for multi-level interventions. At the organizational level, institutions should build resource-enriched environments by adopting high-well-being unit models, which involve optimizing shift scheduling to ensure adequate rest, designing equitable promotion pathways, and establishing compensation systems aligned with job value, as evidenced by the economic security effect observed in nurses earning over 6,000 RMB monthly. High-pressure departments (e.g., emergency units) require routine stress-mitigation measures, including regular team psychological support sessions, post-critical-incident debriefings, and institutionalized compensatory rest periods. At the individual level, targeted training programs should be implemented based on personality profiles: For high-neuroticism nurses, cognitive-behavioral therapy (CBT) workshops for emotional regulation should be prioritized, along with placements in moderate-stress roles supported by mentorship. For high-openness/conscientiousness nurses, engagement in process optimization, innovative technology development, and mentorship roles can transform personal strengths into organizational assets. Additionally, universal active coping skill modules should be developed, featuring standardized training curricula for all nurses. These modules should systematically enhance team-wide psychological resilience through structured problem-solving techniques, effective communication and help-seeking strategies, time management training, and positive psychology exercises such as gratitude journaling and strength identification (24).

To ensure scientific rigor, we must conscientiously acknowledge the limitations of this study, which serve as critical foundations for future research with greater depth and scope. This approach not only upholds scholarly responsibility but also advances knowledge in the field. First, the cross-sectional design inherently limits causal inference. While the verified mediation model reveals inter-variable correlations, it cannot establish deterministic causal chains. For instance, although the model suggests personality influences well-being, reverse causality remains plausible—prolonged low occupational well-being might conversely erode positive personality traits, fostering pessimism. Interpretation of findings should maintain this caution. Second, reliance on self-reported questionnaires introduces two systemic biases: Common method variance (CMV) potentially inflates correlations, as all variables were measured through a single instrument. Social desirability bias may lead nurses to subconsciously provide “positive” or socially expected responses regarding well-being and stress coping, despite anonymous data collection protocols. Third, the homogeneity of our sample—exclusively comprising practicing nurses from Lanzhou City, Gansu Province, China—constrains external validity. Generalization to nurses in other cultural contexts, regions, or healthcare systems requires extreme caution, with applicability likely restricted to populations sharing comparable cultural and institutional characteristics. Fourth, while our mediation analysis prioritized literature-established core variables (controlling for age, gender, and department) and adhered to parsimony principles and Bootstrap sample requirements, omitted confounders may distort pathway estimates. Critical unmeasured variables include: Department-specific stressors: Nurses in intensive care units (ICUs) face distinct high-intensity, high-mortality environments requiring fundamentally different coping resources compared to general ward nurses (25). Professional seniority: Novice nurses experience transitional shocks absent in experienced counterparts who possess richer skills and social resources (26). Future research should validate the stability of pathways through hierarchical mediation modeling.

Building on critical reflections of existing research, we propose specific and actionable directions to advance a more comprehensive, dynamic, and nuanced understanding of these mechanisms. First, longitudinal studies tracking newly recruited nurses over 3–5 years should be prioritized to observe the interactions and evolution of personality traits, coping styles, and occupational well-being during their transition from initial “transition shock” to professional adaptation. Such designs would strengthen causal inferences about temporal relationships. Second, mixed-methods approaches integrating quantitative analysis with qualitative interviews should be adopted. While standardized questionnaires provide generalizable patterns, in-depth interviews can uncover nurses’ personalized interpretations of well-being and the contextual rationales behind their coping choices, thereby enriching mechanistic explanations. Third, multilevel models must account for nested determinants of well-being by simultaneously incorporating individual-level factors (e.g., personality, coping strategies), team-level dynamics (e.g., leadership styles, peer support), and organizational-level policies (e.g., hospital culture, staffing norms) through hierarchical linear modeling (HLM). This approach disentangles the complex interplay of influences across systemic layers. Fourth, extending this framework to physicians, physiotherapists, and other frontline healthcare workers through comparative studies would test the universality of identified mechanisms while informing tailored mental health support systems for diverse medical professions. Finally, international collaborative efforts to replicate findings across cultural contexts are essential. Cross-cultural validations will clarify whether personality-driven pathways represent universal psychological processes or are modulated by cultural norms, ultimately deepening our understanding of culture’s role in shaping occupational mental health. Collectively, these directions transform isolated observations into an integrative paradigm that bridges individual adaptability with systemic resilience.

In conclusion, this large-scale empirical study unveils the core mechanisms linking nurses’ personality traits to occupational well-being. Conscientiousness positively correlates with active coping strategies, creating a self-reinforcing cycle where personality strengths foster effective stress management, resource acquisition, and sustained well-being enhancement. Conversely, neuroticism indirectly diminishes well-being through passive coping, yet its pathway contributes minimally (<5.69%), highlighting a stark efficacy asymmetry between proactive and reactive strategies. These findings not only elucidate the dynamic psychological adaptation of nurses through the lens of Conservation of Resources (COR) theory but also establish the innovative Personality-Coping Asymmetry Model, advancing theoretical foundations in occupational health psychology. Practically, intervention systems should prioritize: (1) optimizing stress-buffering mechanisms for high-intensity departments and enhancing compensation equity; (2) implementing personality-tailored empowerment programs—delivering CBT-based emotional regulation training for neuroticism-prone nurses while creating process improvement opportunities for open/conscientious individuals; (3) developing standardized active coping curricula to systematically strengthen psychological resilience across nursing teams. Future research must transcend current limitations through longitudinal designs to establish causal chains and cross-cultural comparative studies to validate mechanism universality. Ultimately, this trajectory will contribute to building sustainable professional ecosystems that safeguard nurse well-being—a critical step toward preserving this invaluable healthcare workforce and ensuring systemic sustainability.

## Data Availability

The raw data supporting the conclusions of this article will be made available by the authors, without undue reservation.
